# Clinical Considerations for Return to Driving a Car following a Total Knee or Hip Arthroplasty: A Systematic Review

**DOI:** 10.1155/2020/8921892

**Published:** 2020-07-06

**Authors:** Annalisa Na, Kacy Richburg, Zbigniew Gugala

**Affiliations:** ^1^Division of Rehabilitation Sciences, University of Texas Medical Branch, Galveston, TX 77555-0165, USA; ^2^Department of Orthopaedic Surgery and Rehabilitation, University of Texas Medical Branch, Galveston, TX 77555-0165, USA

## Abstract

**Aim:**

The purpose of this study is to systematically review patient characteristics and clinical determinants that may influence return to driving status and time frames following a primary TKA or THA and provide an update of the current literature.

**Methods:**

This review was completed per the Preferred Reporting Items for Systematic reviews and Meta-Analyses (PRISMA) guidelines. Final electronic database searches were completed in October 2019 in Medline/PubMed, Medline/OVID, Cumulative Index to Nursing and Allied Health Literature (CINAHL), and Cochrane Library using preselected search terms. Manuscripts of prospective and nonrandomized studies that examined the return to driving a car after a primary knee or hip arthroplasty patients were included. The Methodological Index for Non-Randomized Studies was used to measure study quality. Two authors selected studies and assessed their qualities. All disagreements were resolved through discussion and, as needed, a third reviewer. Data on study title, author(s), country, year, study design, sample size, inclusion and exclusion criteria, age, BMI, gender, statistical analyses, driving measure, follow-up time, surgical approach, laterality, and postoperative management were extracted from each study.

**Results:**

A total of 23 studies were eligible, including 12 TKA studies (*n* = 654) with mean ages between 43 and 82 years, 9 THA studies (*n* = 922) with mean ages between 34 and 85 years, and 2 combined TKA and THA (TKA, *n* = 815; THA, *n* = 685), yielded MINORS scores between 6 and 12. Most patients achieved or exceeded preoperative response times between 1 and 8 weeks following a TKA and 2 days to 8 weeks following a THA, and/or self-reported return to driving between 1 week and 6 months. Influences on return to driving time included laterality and pain, but gender was mixed. *Discussion*/

**Conclusions:**

Study results were consistent with previous systematic reviews in that return to driving a car after a primary TKA or THA is highly variable, and most commonly occurs around 4 weeks, but can range between 2 and 8 weeks. While various patient and clinical factors can influence return to driving for a TKA or THA, the most common contributing facts were pain and laterality. The heterogeneous nature of the studies prevented a meta-analysis for determining contributions of return to driving following a primary TKA or THA. Regardless, this study updates previous systematic reviews and presents insight on patient and clinical factors beyond generalized timeframes for return to driving a car. This information and results from future studies are essential to guide clinical recommendations and patient and clinician expectations for return to driving a car after a primary TKA or THA.

## 1. Introduction

The incidence of primary total knee arthroplasty (TKA) and total hip arthroplasty (THA) as treatment of severe osteoarthritis (OA) is increasing due to the aging population [1]. The USA prevalence of knee OA increased from 14% at age of 25-60 years to 37% at age of 60 years and older [2]. In the 1970s, TKA rates trended at 31.2 per 100,000 person-years and THAs at 50.2. Approximately 30 years later, in the 2000s, TKAs increased to 220.0 per 100,000 person-years while THAs increased to 145.5 [3]. Much of the TKA and THA growth in patients with severe OA can be attributed to the advancement in technology and notable postoperative rehabilitation improvements [4]. Despite surgical and recovery success, the independence and quality of life of patients receiving a TKA or THA could be attenuated if their return to driving a car is delayed [5].

Recommendations for patients to return driving a car after a TKA and THA must identify the optimal period that balances safety and quality of life, which is difficult to determine. The American Academy of Orthopaedic Surgeons suggests that: *“… it is safe to resume driving when you are no longer taking narcotic pain medication, and when your strength and reflexes have returned to a more normal state. Your doctor will help you determine when it is safe to resume driving”* [5]. Unfortunately, such recommendations are not associated with objective measures, do not provide clear guidelines, and place an undue burden on clinicians. Recent studies have attempted to identify common postoperative timelines for return to driving a car following a primary TKA or a THA.

Previous studies established return to driving a car time after a TKA or THA based on driving simulator response times. Normalizing pre- and postoperative response times on driving simulators is the current reference standard but is also a surrogate measure to identify the necessary strength and reflexes [5] for driving a car [6–8]. Patients achieving preoperative response times on a driving simulator after a TKA or a THA are considered ready to return to driving their cars [6–8]. Several previous reviews have published time frames for return to driving a car after a TKA or THA based on normalized response times [6–8], which is beneficial for clinics without driving simulators. In 2016, van der Velden et al. [8] published meta-analysis on brake response times and found that preoperative times are achieved postoperatively in 2 weeks for a right-sided THA and 4 weeks for a right-sided TKA in countries with right-side drive vehicles (e.g., the USA). In 2018, Hartman et al. [6] completed a systematic review to find that preoperative brake response, movement, and reaction times were achieved postoperatively at 2 weeks for a left-sided TKA and 4 weeks for a right-sided TKA in studies from countries with right-side drive cars. While these reviews provided valuable timeline recommendations on return to driving a car following a TKA or THA, they concluded that additional patient and surgical factors need to be considered [6–8].

The previous published reviews [6–8] did not systematically examine the highly variable patient and surgical factors that can influence the return to driving a car timeline after a TKA or THA. A recent study found that patient-perceived readiness, joint range of motion, patient demographics, pain, surgical procedure, and postoperative management strategies can influence return to driving a car [9]. However, previous reviews did not account for these variabilities, nor include studies published after 2016 [6, 8]. Therefore, an updated systematic review that includes these confounding variables is warranted. The purpose of this systematic review is to examine characteristics of patient, driving, and clinical determinants that may influence return to driving a car and time frames following a primary TKA or THA. Examination of such confounding variables is important to help clinicians make informed and patient-specific recommendations for return to driving a car after a TKA or THA.

## 2. Materials and Methods

A comprehensive literature search was performed for this systematic review according to the Preferred Reporting Items for Systematic reviews and Meta-Analyses (PRISMA) guidelines.

### 2.1. Data Sources

Electronic database searches were completed in Medline/PubMed, Medline/OVID, Cumulative Index to Nursing and Allied Health Literature (CINAHL), Cochrane Library, New York Academy of Medicine's Grey Literature Report, and Google Scholar. Sources were also found by scanning references of pertinent articles to ensure a complete review of all related literature. All searches were completed and finalized in October 2019.

### 2.2. Eligibility Criteria

Eligible studies included peer-reviewed manuscripts (e.g., not magazine article, editorial book chapter, and conference abstract) published in the English language containing prospective study designs, recruited patients receiving a primary TKA or THA who were adults of age 21 years and older, and assessed driving outcome by self-reported, motor vehicle accidents, driver safety, brake reaction time, driver reaction time, or total breaking time using driving simulators. Studies on unicompartmental TKA or patients with revision TKA or THA patients and or patients with other orthopaedic conditions/arthroplasties (e.g., fractures, shoulder arthroplasty, elbow arthroplasty, and ankle arthroplasty) were excluded.

### 2.3. Search Strategy

We used a systematic search strategy for indemnifying studies relevant to the patient population and outcomes of interest. Population terms included “joint arthroplasty,” “hip arthroplasty,” “knee arthroplasty,” and variations where “arthroplasty” was replaced by “replacement.” Outcome terms included “brake time,” “braking,” “reaction time,” “driving,” and “automobile.” Search limits consist of *English* and with *humans*. Medical subject headings (MeSH) and key terms were used when applicable. All findings were imported and managed in Mendeley Version 1.19.2 (Mendeley Ltd, London, UK) bibliographic software.

### 2.4. Study Selection

The titles, abstracts, and full texts of the retrieved studies were independently screened for eligibility by 2 authors. Disagreements were examined and resolved by discussions and, when needed, involved a referee reviewer.

### 2.5. Quality Assessment

The Methodological Index for Non-Randomized Studies (MINORS) was used to examine the quality of each study [10]. The MINORS is a valid and reliable 12-item survey with 8 questions to assess noncomparative surgical studies and 12 to assess comparative surgical studies [10]. The comparative component was not appropriate for the targeted studies and was eliminated. Scoring includes 0 = not-reported, 1 = reported but inadequate, or 2 = reported and adequate [10]. Two authors independently assessed methodological quality, and discrepancies were examined and discussed to achieve a consensus.

### 2.6. Data Extraction

Study data were extracted and recorded using a standardized form. Data extracted included study title, author(s), country, year, study design, sample size, inclusion and exclusion criteria, age, BMI, gender, statistical analyses, driving measure, and follow-up time. Clinical data for all arthroplasty included preoperative status, prosthesis implant type, laterality, mode of anesthesia, surgical approach, use of cement, and postoperative management. We also recorded incision size and patella resurfacing for TKA and femoral component size for THA.

## 3. Results

**Figure 1:**
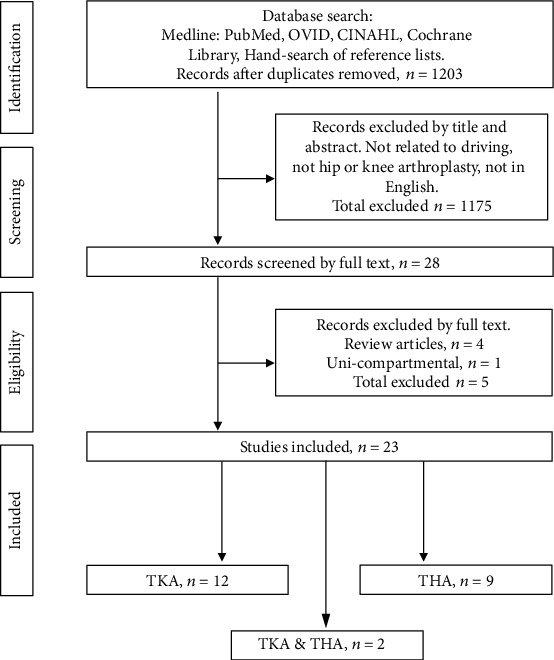
Flow chart of study selection process.

The systematic search yielded 62 records that were screened per the inclusion criteria, resulting in full-text reviews for 25 studies (Figure 1). Of those reviewed, one study was excluded for not having a quantifying measure for return to driving, one study was not in English, one study was on knee osteoarthritis and not TKA, and one study was on unicompartmental TKA. A total of 23 studies met the eligibility criteria (Figure 1).

### 3.1. Methodological Quality

The 2 examiners demonstrated strong agreement with preliminary scoring (interclass correlation coefficient, ICC = 0.91). Final MINORS scores for the 23 studies ranged from 6 to 12 (Table 1). None of the reviewed studies reported methodological blinding, and 6 studies included a priori power calculations.

### 3.2. Population

Of the 23 studies, 12 studies included TKA only cohort, 9 studies included a THA only cohort, and 2 studies included both TKA and THA cohorts.

The TKA studies included a total of 654 patients with reported mean ages ranging between 43 and 82 years (Table 2). Five TKA studies were conducted in the United States (*N* = 397, 60.7%), 3 in Germany (*N* = 85, 13.0%), and 1 in Austria (*N* = 31, 4.7%), United Kingdom (*N* = 29, 4.4%), Ireland (*N* = 98, 1.5%) and Taiwan (*N* = 14, 2.1%). Ten TKA studies reported gender distribution with 391 females or 62.6%. Of the 10 TKA studies, there were 238 right TKAs (60.1%), 145 left TKAs (36.7%), and 13 bilateral TKAs (3.3%).

The THA studies included a total of 922 patients between ages 34 and 85 years. Three THA studies were conducted in the United States (*N* = 412, 44.7%), 3 in the United Kingdom (*N* = 172, 18.7%), 2 in Australia (*N* = 298, 32.3%), and 1 in Germany (*N* = 40, 4.3%). Of the 9 THA studies, complete gender data were missing in 2 studies (*N* = 39); therefore, of 883 patients, 461 or 52.2% were females. There were 463 right THA (63.6%), 250 left THA (34.3%), and 15 bilateral THA (2.1%). Of the 15 patients with bilateral THA, 2 patients were single-staged bilateral THA, and 13 patients had a subsequent contralateral THA.

Two studies reported on a combined TKA and THA cohort both from the United States, with a total of 812 patients receiving a TKA and 685 patients receiving a THA. Of the patients receiving a TKA or THA, 869 (58.1%) were females.

### 3.3. Outcomes

Return to driving was measured using a driving simulator in 17 studies, self-reported outcomes in 4 studies, and both the driving simulator and self-reported in 1 study. Studies reported that most patients returned to driving a car between 1 and 8 weeks following a TKA and 2 days and 8 weeks following a THA (Table 2). Meanwhile, overall self-reported return to driving ranged between 1 week and 6 months in all studies.

### 3.4. Surgical Factors

For TKA, surgical factors included the approach, incision size, cement use, patella resurfacing, prosthesis implant, perioperative anesthesia, and postoperative management which were evaluated to determine the influence on return to driving (Table 3). TKA approaches were highly variable, mostly cemented techniques, with early rehabilitation that focused on weight-bearing and mobilization on postoperative day 1, and return to driving a car ranged between 2 and 6 weeks.

For THA, surgical factors included the approach, femoral size, cement use, prosthesis implant, perioperative anaesthesia, and postoperative care (Table 4). The posterior approach was the most common, as it was in 4 studies with return to driving time frame ranging between 4 and 6 weeks. Return to driving for the anterior approach ranged between 3 and 4 weeks and the lateral approach ranged between 2 days and 4 weeks.

### 3.5. Factors Influencing Return to Driving following a TKA

Gender and age were reported in 7 TKA studies, with 4 reporting that being female and 1 reporting that being older was related to increased time for the return to driving a car (Table 5). Laterality influenced the return to driving in 6 TKA studies but was an insignificant covariate in 2 TKA studies. Other patient factors, including self-perceived readiness and pain, also influenced postoperative return to driving timeframes. Meanwhile, 1 study reported that current assistive device use did not influence return to driving, and 1 study reported that preoperative assistive influenced postoperative driving time frames. Only 1 TKA study examined driving speed as a potential influence on return to driving and found a significant effect; however, no other studies examined the variable.

### 3.6. Factors Influencing Return to Driving following a THA

Of the THA studies, 9 studies examined age and/or gender. Two studies reported that age had no effects, but that being female influenced the time of return to driving a car (Table 6). Laterality influenced return to driving in 3 THA studies but was reported to be an insignificant covariate in 3 other THA studies. Self-perceived readiness was examined and reported to be a significant influence on return to driving in 4 THA studies. Physical function influenced return to driving in 2 THA studies. Assistive device use was not related to return to driving in 2 THA study but significant in another THA study. Manual transmissions in 3 THA studies and pain in 1 THA study did not influence postoperative return to driving a car time frames.

## 4. Discussion and Conclusion

Recommendations for return to driving a car after a TKA and THA are difficult because it must optimize safety and the patient's quality of life. Current studies use surrogate measures, including response times on driving simulators or self-reported questionnaires, to assess return to driving. Previous systematic reviews report that return to driving based on predetermined surrogate measures (e.g., brake response times) occurs at approximately 4 weeks after a right TKA in countries of right-sided driving [7, 8]. However, recent publications after the last systematic searches conducted in 2016 found a reduction in return to driving a car after surgery earlier than 4 weeks. For example, in an updated study in 2019, Dalury et al. [4] reported that patients returned in 2 weeks after surgery. Dalury et al. [14] prior study in 2011 found that returned to driving a car took 4 weeks. The authors attributed the reduced time to the advancement and modernization of surgery and postoperative management [4]. Hence, the current systematic review is essential, because (1) it provides an update of the current literature, including 5 new studies since the last systematic review, and (2) it identified gender, laterality, and pain as factors that can influence return to driving after a TKA or a THA, which have never been systematically examined.

**Figure 2:**
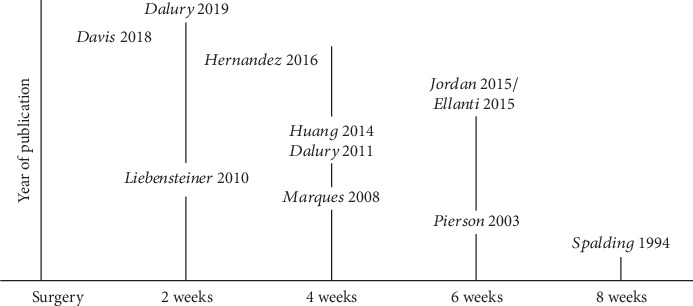
Recommended return to driving for a right-sided total knee arthroplasty.

**Figure 3:**
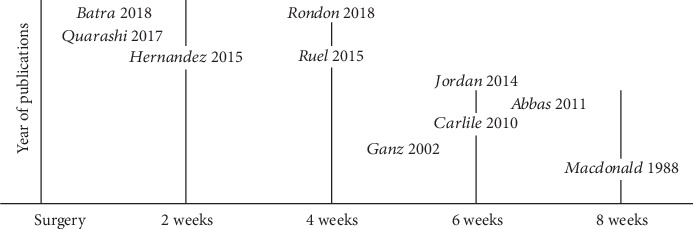
Recommended return to driving for a right-sided total hip arthroplasty. THA studies after 2015 are recommending patients to return driving their cars at 4 weeks or less.

The primary finding of this systematic review was that most patients returned to driving a car at about 2 to 4 weeks after a right-sided TKA or THA in predominately right-sided driving countries, which was overall consistent with previous studies. Hartman et al. [6] completed a systematic review of studies before 2017 and found that postoperative brake response times normalized to preoperative times at 4 weeks with a right TKA and at 2 weeks with a left TKA for right-handed driving. van der Velden et al. [8] completed a meta-analysis on studies of TKA and THA cohorts published before March 2016 and founded brake response times normalized at 4 weeks for right TKA and 2 weeks for R THA. DiSilvestro et al. [7] in 2016 examined studies of orthopaedic surgeries, not exclusive to TKA or THA, published before August 2015, and found return to driving occurred at 4 weeks for patients with right-sided TKA or THA and 1 week for left-sided TKA and THA. While these systematic reviews most commonly reported 4 weeks, it included studies that had recommendations between 2 and 8 weeks. Besides the 5 new studies, the current systematic review examined several similar studies and observed an interesting trend. Specifically, publications from 2016 or later reported return to driving a car between 1 and 4 weeks after a TKA (Figure 2) or a THA (Figure 3). While more studies are needed, there appears to be a trend of older studies reporting longer times before return to driving a car and newer studies reporting shorter times. Nevertheless, given the strong variability and changes in TKA and THA practices over a short period, continued assessment of return to driving a car after surgery needs to be frequent.

Making conclusive recommendations or completing a meta-analysis is difficult due to the heterogeneity of studies examining the return to driving a car following a TKA or THA. Therefore, we followed suit as DiSilvestro et al. [7] and Hartman et al. [6] chose a systematic review and forgo a meta-analysis. Such a systematic review allowed us to examine confounding factors that can influence return to driving time.

Results of the current study found that in addition to laterality, pain was a contributing factor for when patients return to driving following a TKA or THA and gender yielded mixed results. Unfortunately, pain was assessed in 3 TKA studies and no THA studies; however, none of these studies reported a lack of relationship between pain and time for return to driving a car. Pain can reduce muscle activation that inhibits response times [32] needed for driving reactions, braking reactions, or achieve force thresholds for emergency braking. Future studies should consider pain levels, especially since narcotic use for pain management while driving can jeopardize societal and patient's safety. Therefore, it is necessary to determine the influence of pain on driving to determine adequate pain management and return to driving. In contrast, gender was examined in several studies; however, the results were mixed. Specifically, 57% of TKA studies and 22.2% of THA studies reported that being female was associated with a slower return to driving a car. The studies that reported gender to effect return to driving time also reported that preoperative response times were slower for females than males [15, 28], while the other studies that reported on significant effects of gender assessed return to driving using self-reported measures [9, 11, 27]. Therefore, these results make it difficult to discern if the effects of gender on time of returning to drive a car after a TKA or THA are due to psychosocial factors versus actual readiness to drive.

This study is with several limitations. The studies examined lacked homogeneity, which limited the ability to examine patient and clinical factors that influence return to driving following a TKA or THA. While the driving simulator is the current reference standard to determine return to driving a car after surgery, its relationship to actual motor vehicle accidents in patients with a TKA or THA is unclear, and it does not account for self-confidence, efficacy, and potentially other psychosocial issues. Specifically, the minimum response times that yield safe versus unsafe drivers following a TKA or a THA have never been validated. Further, this systematic review only found 1 study that examined postoperative motor vehicle accidents, but this study did not correlate these values to response times on driving simulators. Combining self-perceived readiness and simulator testing results allows clinicians to consider important subjective and objective data. Only 1 study examined both outcomes; Hernandez et al. [18] reported that while a majority of patients who achieved pre-THA response times on the simulator also self-reported readiness to drive, there was a subgroup population where the outcomes did not align. It is important to note that these factors are determined by driving simulators and achieving preoperative levels measured in the presence of arthritic signs and symptoms. Preoperative values are slower than age- and gender-matched values reported in the literature and can be influenced by arthritis and other comorbidities that were not assessed in the reviewed studies [33]. Unfortunately, the validity of simulator response time and actual driving safety are difficult to test; however, careful considerations are needed to determine appropriate response times for return to driving. Last, not enough studies reported clinical and surgical determinants that influence return to driving a car for conclusions or trends to be concluded.

Future studies with greater number of subjects need to examine self-perceived readiness drive, objective data, and specific details on surgical and clinical management.

Nevertheless, actual potential covariates that may contribute to return to driving durations have never been systematically examined, and this study determined that not only was laterality but also factors such as gender, testing techniques, and pain levels need to be considered. Last, this study provides an update on return to driving following a TKA or THA in publications before 2020 and observed a trend of reduction in return to driving times after surgery. Additional and ongoing research is needed as research and clinical practice advances for primary TKA and THA.

## Figures and Tables

**Table 1:** Quality scoring studies using the Methodological Items for Non-Randomized Studies (MINORS) criteria.

Study	Aim	Patients	Data	Endpoint	Blinding	Follow-up	<5% loss	Power	MINORS score
Abbas et al. [11]	1	2	2	1	0	1	0	0	7
Batra et al. [12]	2	2	1	1	0	2	2	0	10
Carlile et al. [13]	2	0	2	2	0	2	2	0	10
Dalury et al. [14]	2	0	2	2	0	2	2	0	10
Dalury et al. [4]	2	0	2	2	0	2	0	0	8
Davis et al. [15]	1	2	2	2	0	2	1	2	12
Ellanti et al. [16]	2	0	1	2	0	2	1	0	8
Ganz et al. [17]	2	0	2	2	0	2	2	0	10
Hernandez et al. [18]	2	0	2	2	0	2	2	2	12
Hernandez et al. [19]	1	1	2	2	0	2	2	2	12
Huang et al. [20]	2	1	2	2	0	1	0	2	10
Jordan et al. [21]	1	2	2	2	0	2	1	0	10
Jordan et al. [22]	2	2	2	2	0	2	1	0	11
Liebensteiner et al. [23]	2	2	2	2	0	2	1	0	11
MacDonald et al. [24]	2	2	2	2	0	2	1	0	11
Marques et al. [25]	2	2	2	1	0	1	1	0	9
Marques et al. [26]	2	0	2	2	0	2	2	0	10
Muh et al. [27]	1	2	1	1	0	2	2	0	9
Pierson et al. [28]	2	0	2	2	0	2	0	0	8
Qurashi et al. [29]	2	2	2	2	0	1	2	2	13
Rondon et al. [9]	2	2	0	2	0	0	0	0	6
Ruel et al. [30]	2	2	2	2	0	2	0	2	12
Spalding et al. [31]	2	0	2	2	0	2	1	0	9

Each item is scored with 0 = not reported, 1 = reported but inadequate, or 2 reported and adequate. MINORS score was determined as the sum of the items.

**Table 2:** Summary of studies.

Study	Population	Study design	Participants	Measurement	Time for return to driving
Dalury et al. [14] (USA)	R TKA only	Prospective cohort	*n* = 29; age = 66 years; females = 18; BMI = NR	Driving simulator	4 weeks
Dalury et al. [4] (USA)	R TKA only	Prospective cohort	*n* = 40; age = 69.1 years; females = 30; BMI = 29.3 kg/m^2^	Driving simulator	2 weeks
Davis et al. [15] (USA)	Unilateral TKA	Prospective cohort	*n* = 32; *R* = 19; *L* = 13; age = 62.6 years; females = 19; BMI = NR	Driving simulator	1 to 2 weeks
Ellanti et al. [16] (Ireland)	Unilateral TKA	Prospective cohort	*n* = 38; *R* = 43; *L* = 55; age = 59.5 years; females = 54; BMI = NR	Self-report	6 weeks
Hernandez et al. [18] (USA)	R TKA only	Prospective cohort	*n* = 47; age = 67.5 years; females = 29; BMI = 32.5 kg/m^2^	Driving simulator	2 to 4 weeks
Huang et al. [20] (Taiwan)	R TKA only	Prospective cohort	*n* = 14; age = 63.14 years; females = 10; BMI = NR	Driving simulator	4 weeks
Jordan et al. [21] (Germany)	Unilateral TKA	Prospective cohort	*n* = 40; *R* = 20, median age = 69 years; *L* = 20, median age = 74 years; females = 22; BMI = NR	Driving simulator	*R* = 6 weeks *L* = 8 days
Liebensteiner et al. [23] (Austria)	Unilateral TKA	Prospective cohort	*n* = 31; *R* = 13; *L* = 18; age = 65.7 years; females = 18; BMI = NR	Driving simulator	2 weeks
Marques et al. [25] (Germany)	R TKA only	Prospective cohort	*n* = 21; age = 69.1 years; females = 12; BMI = NR	Driving simulator	30 days
Marques et al. [26] (Germany)	L TKA only	Prospective cohort	*n* = 24; age = 63.2 years; females = 11; BMI = NR	Driving simulator	10 days
Pierson et al. [28] (USA)	TKA	Prospective cohort	*n* = 3; *R* = 12; *L* = 6; *B* = 13; age = 68.6 years; females = 14	Driving simulator	6 weeks
Spalding et al. [31] (UK)	Unilateral TKA	Prospective cohort	*n* = 29; *R* = 12, *L* = 6; age = 74 years; females and BMI = NR	Driving simulator	8 weeks
Abbas et al. [11] (UK)	Unilateral THA	Prospective cohort	*n* = 130; *R* = 85; *L* = 45; age range = 39 to 80 years; females = 50; BMI = NR	Self-report	6 to 8 weeks
Batra et al. [12] (Australia)	THA	Prospective cohort	*n* = 198; *R* = 95; *L* = 89; *B* = 14; age = 69 years; females = 129; BMI = 28.10 kg/m^2^	Self-report	1 week
Carlile et al. [13] (UK)	THA	Prospective cohort	*n* = 20; *R* = 15; *L* = 5; age = 69 years; females = 9; BMI = NR	Driving simulator	6 weeks
Ganz et al. [17] (USA)	Unilateral THA	Prospective cohort	*n* = 90; *R* = 52; *L* = 38; age = 68.9 years; females = 34; BMI = NR	Driving simulator	4 to 6 weeks
Hernandez et al. [19] (USA)	R THA only	Prospective cohort	*n* = 38; age = 62 years; female and BMI = NR	Driving simulator and self-report	2 weeks
Jordan et al. [22] (Germany)	Unilateral THA	Inception cohort	*n* = 40; *R* = 20; *L* = 20; age = 66.6 years; females = 15	Driving simulator	*R* = 6 weeks *L* = 8 days
MacDonald et al. [24] (UK)	Unilateral THA		*n* = 25; *R* = 12, age = 61 years; *L* = 9, age = 58; females = 6	Driving simulator	8 weeks
Qurashi et al. [29] (Australia)	THA	Prospective	*n* = 100; *R* = 56; *L* = 44; age = 62.9; females = 50	Driving simulator	2 days
Ruel et al. [30] (USA)	R THA only	Prospective	*n* = 90′ at 2 weeks: *n* = 30, age = 62.5 years; females = 21; at 3 weeks: *n* = 29; age = 62.45 years, females = 11; at 4 weeks: *n* = 31; age = 64.14, females = 16	Driving simulator	4 weeks
Muh et al. [27] (USA)	TKA and THA	Retrospective	TKA = 258; age = 70.1, females = 185 THA = 194; age = 70.5; females = 120	Self-report	≤6 months
Rondon et al. [9] (USA)	TKA and THA	Prospective	TKA = 554; age = 66.0, BMI = 31.1 kg/m^2^; females = 327 THA = 490; age = 63.5; BMI = 29.03 kg/m^2^; females = 237	Self-report	3 to 4 weeks

TKA: total knee arthroplasty; THA: total hip arthroplasty; BMI: body mass index; R: right; L: left; NR: not reported.

**Table 3:** TKA summary of surgical procedures.

Study	Approach	Incision	Cemented	Patella resurface	Prosthesis	Anesthesia	Postoperative care
Dalury et al. [14]	Limited extensor mechanism with cruciate retaining	10-14 cm	Yes	Yes	Sigma TKA	96.6% spinal 3.4% general	Day 1 = WBAT with 5, 30 min acute PT sessions progress from walker to cane and ROM; day 3 = discharge to home or inpatient facility
Dalury et al. [4]	Tricompartment with trivector	—	Yes	—	—	Spinal	Day 1 = out of bed and PT
Davis et al. [15]	Midvastus with cruciate retaining	Mean = 9.8 cm	Yes	Yes	NexGen® complete knee solution	Spinal	After epidural removal, start PT. discharge to outpatient PT
Ellanti et al. [16]	Medial parapatellar with cruciate retaining	—	Yes	No	—	Spinal	Day 1 = full WB with PT.
Hernandez et al. [19]	Midvastus	—	—	—	—	Spinal	Day 1 = mobilization with PT.
Huang et al. [20]	Medial parapatellar	9 to 12 cm	—	—	Zimmer LPS-flex mobile	—	Hospital PT walking with walker and CPM. Discharge to home with exercise, no PT.
Jordan et al. [22]	—	—	—	—	Genesis II	—	—
Liebensteiner et al. [23]	Medial parapatellar	—	Yes	—	Kinemax, Stryker	—	CPM, ROM, then outpatient PT.
Marques et al. [25]	—	—	—	—	Rotational = 33.3% Bicondylar = 66.7%	—	Day 2 = WBAT gait training
Marques et al. [26]	—	—	—	—	Rotational = 20.8% Bicondylar = 75.0% Unicondylar = 4.2%	—	Day 1 = PT in bed Day 2 = WBAT gait training
Pierson et al. [28]	Medial parapatellar	—	Yes	Yes	—	—	Day 1 = WBAT with device as needed

ROM: range of motion; WBAT: weight bearing as tolerated; PT: physical therapy; CPM: continuous passive motion.

**Table 4:** THA summary of surgical procedures.

Study	Approach	Femoral size	Cemented	Prosthesis	Anesthesia	Postoperative care
Abbas et al. [11]	Posterior	—	—	—	—	Precautions; day 1 = WBAT gait training
Batra et al. [12]	Anterior bikini	28, 32, and 36 mm	Both	Smith and nephews	—	PT
Carlile et al. [13]	Stemmed prosthesis	—	—	—	—	—
Ganz et al. [17]	Posterior	—	Cemented or hybrid	—	Spinal	Precautions; day 1 = partial to WBAT; cane at discharge
Hernandez et al. [18]	Muscle sparing	Modern press-fit	—	—	Spinal	Rapid mobilization
Jordan et al. [21]	Transgluteal	—	Hybrid	—	—	Highly variable
Qurashi et al. [29]	Lateral with SuperPATH	—	No	Metal or ceramic on polyethylene	—	No precautions; PT
Rondon et al. [9]	Anterior = 57.3%; lateral = 28.4%; posterior = 14.3%	—	—	—	—	PT until discharge
Ruel et al. [30]	Posterior	—	—	—	—	Day 1 = ambulate with walker. PT and progression

WBAT: weight-bearing as tolerated; PT: physical therapy.

**Table 5:** Factors influencing return to driving following a total knee arthroplasty.

Study	Measure	Gender	Age	Readiness^∗^	Pain	AD	Function	Laterality	Other
Dalury et al. [14]	1. Gas-off time	No	No	—	—	—	—	—	—
	2. Transition time	No	No	—	—	—	—	—	—
	3. Reaction time	No	No	—	—	—	—	—	—
Davis et al. [15]	1. Brake response time	Yes	No	—	—	—	—	No	—
Ellanti et al. [16]	1. Self-reported	No	No	—	Yes	—	—	Yes	Driving status—yes^
Hernandez et al. [19]	1. Brake response time	No	No	Yes	Yes^	No	—	—	BMI—no
Huang et al. [20]	1. Total braking time	—	—	—	—	—	Yes	—	Driving speed—yes
Jordan et al. [22]	1. Brake response time	—	—	—	—	—	—	Yes	—
	2. Brake force	—	—	—	—	—	—	Yes	—
Liebensteiner et al. [23]	1. Brake response time	—	—	—	—	—	—	Yes	—
Muh et al. [27]	1. Self-reported accidents	Yes	Yes	—	—	—	—	—	—
Pierson et al. [28]	1. Brake response time	Yes	—	—	—	—	—	No	—
Rondon et al. [9]	1. Self-reported	Yes	No	Yes	Yes	Yes^	Yes	Yes	Inpatient PT, anemia^, and manual—yes; BMI—no
Spalding et al. [31]	1. Total reaction time	—	—	—	—	—	—	Yes	—

AD: assistive device; BMI: body mass index; —, not reported; ^∗^self-reported readiness to drive; ^preoperative status.

**Table 6:** Factors influencing return to driving time following a total hip arthroplasty.

Study	Measure	Gender	Age	Readiness^∗^	AD	Function	Laterality	Other
Abbas et al. [11]	1. Self-reported	Yes	—	Yes	—	—	No	Manual—no
Batra et al. [12]	1. Self-reported	—	—	Yes	No	Yes	No	Manual—no
Carlile et al. [13]	1. Driving response time	No	—	—	—	—	Yes	—
Ganz et al. [17]	1. Brake response time	No	—	—	—	—	No	Driving patterns—no^
Hernandez et al. [18]	1. Brake response time	No	No	—	No	—	—	—
	2. Self-reported confidence	No	No	Yes	—	—	—	—
Jordan et al. [21]	1. Reaction time	No	No	—	—	—	Yes	Mile per year, transmission, and pain medication—no
	2. Movement time	No	No	—	—	—	Yes	
	3. Brake response time	No	No	—	—	—	Yes	
	4. Maximum brake force	No	No	—	—	—	Yes	
Muh et al. [27]	1. Self-reported accidents	No	No	—	—	—	—	—
Rondon et al. [9]	1. Self-reported	Yes	No	Yes	Yes^	Yes	Yes	ROM—yes; pain—no
Ruel et al. [30]	1. Brake response time	—	Yes	—	—	—	—	—

AD: assistive device; ROM: range of motion; —, not reported; ^∗^self-reported readiness to drive, ^preoperative status.
